# Effect of Methadone or Hydromorphone on Cardiac Conductivity in Dogs Before and During Sevoflurane Anesthesia

**DOI:** 10.3389/fvets.2020.573706

**Published:** 2020-09-24

**Authors:** Stephanie Keating, Ryan Fries, Katherine Kling, Lynelle Graham, Stuart Clark-Price, David J. Schaeffer

**Affiliations:** ^1^Department of Veterinary Clinical Medicine, College of Veterinary Medicine, University of Illinois, Urbana, IL, United States; ^2^Department of Molecular Biomedical Sciences, College of Veterinary Medicine, North Carolina State University, Raleigh, NC, United States; ^3^Department of Clinical Science, College of Veterinary Medicine, Auburn University, Auburn, AL, United States

**Keywords:** methadone, hydromorphone, sevoflurane, QT interval, ECG, dog

## Abstract

**Objective:** To evaluate changes in electrocardiogram (ECG) variables in healthy dogs receiving either methadone or hydromorphone IV before and during sevoflurane anesthesia.

**Study Design:** Prospective clinical study.

**Animals:** Forty client-owned dogs.

**Methods:** Dogs were randomized to receive methadone 0.5 mg/kg IV or hydromorphone 0.1 mg/kg IV in each part of a two-part study. In part one, dogs received the opioid prior to sevoflurane anesthesia (groups MS, *n* = 12 and HS, *n* = 12). Anesthesia was induced with propofol IV, maintained with sevoflurane, and dogs were mechanically ventilated. Standard 6-lead ECG recordings were obtained before opioid administration, 2, 5, and 10 min after opioid administration prior to anesthesia, and during anesthesia 15 min after end-tidal sevoflurane stabilized at 2.4%. In part two, conscious dogs received the same opioid treatments and ECGs were obtained at equivalent time points without undergoing anesthesia (groups M, *n* = 8 and H, *n* = 8). Values for ECG variables were determined by a blinded cardiologist and included: Heart rate (HR), PR interval, QT interval, and HR corrected QT interval (QT_c_) using the Bazett (QT_cB_), Fridericia (QT_cF_), and Van de Water (QT_cV_) formulas. Differences over time and between all four groups were evaluated using ANOVA for repeated measures with significance set at *p* ≤ 0.05.

**Results:** Both methadone and hydromorphone administration reduced HR and prolonged PR and QT intervals, with greater changes observed during sevoflurane anesthesia. The greatest prolongation in QT interval was observed in dogs administered methadone during sevoflurane anesthesia.

**Conclusions and Clinical Relevance:** Methadone and hydromorphone caused disturbances in myocardial electrical activity, and the addition of sevoflurane enhanced these disturbances. Both drugs caused considerable QT interval prolongation into the proarrhythmogenic range, with methadone causing greater prolongation.

## Introduction

Opioid agonists are routinely administered for perioperative analgesia in dogs. Methadone is unique among opioids, acting as a full mu opioid agonist and an antagonist at the N-methyl-D-aspartate (NMDA) receptor, as well as inhibiting the re-uptake of norepinephrine and serotonin within the brain and spinal cord (1, 2). In addition to the multimodal analgesic benefits of methadone, there are fewer undesirable effects, such as vomiting and excitatory behavior, compared to other full mu agonists (3, 4). These advantages have led to the use of methadone for premedication prior to general anesthesia in dogs and cats, particularly in patients experiencing vomiting, chronic or neuropathic pain (3, 5, 6).

Despite the advantages of methadone, there are reports of disturbances in cardiac conduction following oral and intravenous administration in humans (7, 8). The most commonly reported electrocardiographic (ECG) change is prolongation of the QT interval, resulting from prolonged myocardial repolarization. While this change itself may be of little consequence, it allows for early after depolarizations that can ultimately trigger fatal arrhythmias, such as torsades de pointes (9). While QT interval prolongation and associated arrhythmias have predominantly been reported in people taking long term oral methadone for opioid addiction, QT prolongation has also been identified after intravenous methadone administration in hospitalized cancer patients, while morphine administration was not associated with this effect (7).

In addition to QT interval prolongation, methadone has been reported to induce sinus bradycardia in people (10). This effect is suspected to result not only from the mu opioid agonist effects, but also calcium channel blockade, as methadone has a very similar chemical structure to verapamil, a commonly used calcium channel blocker (11, 12). Second and third degree atrioventricular (AV) block has been occasionally noted following IV methadone administration in anesthetized dogs at the University of Illinois Veterinary Teaching Hospital; however, these observations currently remain anecdotal and the effect of methadone on cardiac conduction, and any potentiating effects of inhalational agents on the arrhythmogenic potential of methadone, have not yet been reported in veterinary species.

Hydromorphone is a clinical alternative to methadone that may be selected due to lower cost and greater clinician familiarity, but can induce emesis and lacks the NMDA activity of methadone (13). Hydromorphone may also cause reductions in heart rate (HR) through enhanced vagal activity (14), but its effect on QT interval alteration has not yet been evaluated in healthy dogs. The objectives of the current study were to evaluate ECG variables in healthy conscious dogs before and after the administration of either methadone or hydromorphone IV, and during subsequent sevoflurane anesthesia. It was hypothesized that both opioids would cause reductions in HR, with methadone having a greater effect on QT interval.

## Materials and Methods

### Animals

Forty client-owned dogs were enrolled in the study. Inclusion criteria consisted of dogs between the ages of 0.5–5 years old undergoing anesthesia for elective procedures (dental prophylaxis, ovariohysterectomy, or orchiectomy), and considered to be in good health based on history, physical exam, thoracic auscultation, and routine blood work. Exclusion criteria included irregular heart rhythm, anxious or fractious temperament, administration of medications other than routine heartworm or parasite preventatives, and any pre-existing condition deemed to be painful. The study was approved by the Institutional Animal Care and Use Committee of The University of Illinois (protocol #16187) and written client consent was obtained before enrollment.

### Study Design

The study was a prospective, randomized clinical study that was carried out in two phases due to limitations in opioid supply. Phase one of the study was conducted to evaluate changes in ECG variables over time in dogs administered methadone or hydromorphone IV prior to and during subsequent sevoflurane anesthesia (groups MS and HS, respectively). A sample size calculation was performed, determining that 7 dogs per group with a total of 2 groups would be required to detect a QT interval increase of 20 ms using an alpha value of 0.05 with a power of 80% and a standard deviation of 12 milliseconds (ms) (15). Twelve dogs were enrolled per group to account for possible attrition and variability in the study population. An effect size of 20 ms was selected, as this was considered clinically significant based on the 2005 U.S Department of Health and Human Services industry guidelines. Randomization was achieved using an online random number generator (www.random.org).

In phase two of the study, methadone and hydromorphone were similarly administered without the addition of sevoflurane (groups M and H, respectively) to serve as control groups, with ECG variables obtained at the same time points as phase one. Phase two was conducted to determine if changes in ECG variables following the administration of sevoflurane in phase one were attributable to the additive cardiac depressant effects of sevoflurane or simply ongoing changes over time. Effect size and standard deviation values from phase one were used for sample size calculations in phase two to more accurately predict a sample size that would provide appropriate power while minimizing animal enrollment. Calculations determined that 6 dogs per group with a total of 2 groups would be required to detect a QT interval difference of 50 ms between dogs undergoing sevoflurane anesthesia and conscious dogs at the same time point, with an alpha value of 0.05, a power of 80% and a standard deviation of 31 msec. Eight dogs were enrolled per group for possible attrition or a smaller than expected effect size. Randomization was performed as in phase one.

### Experimental Protocol

Once enrolled in either phase of the study, dogs were randomly assigned to receive either methadone 0.5 mg/kg or hydromorphone 0.1 mg/kg IV. Following treatment assignment, a 20-gauge over the needle catheter (BD Insyte intravenous [IV] catheter, BD Medical, Franklin Lakes, NJ) was placed in the cephalic vein following aseptic preparation, and 1 mL of venous blood was obtained for routine pre-anesthetic blood work. Following a minimum rest period of 10 min, dogs were placed in right lateral, gently restrained if necessary, and a baseline 6-lead ECG recording was obtained (MAC 2000, GE Medical Systems Information Technologies Inc., WI, USA). Following the baseline ECG recording, the randomly assigned opioid treatment was administered as a single IV bolus over 5 s via the catheter and flushed with 3 mL of physiologic saline. Dogs remained in right lateral recumbency for the duration of the experimental period.

For dogs in phase one (groups MS and HS), additional ECG recordings were obtained at baseline, 2, 5, and 10 min post-administration. Anesthesia was then induced with propofol (PropoFlo, Abbott, North Chicago, IL) administered to effect to allow orotracheal intubation, and dogs were transferred to sevoflurane delivered in oxygen to maintain anesthesia. A balanced electrolyte solution (Lactated Ringer's injection, Hospira, Lake Forest, IL) was administered at 5 mL/kg/h and mechanical ventilation was instituted immediately to maintain end-tidal CO_2_ (ETCO_2_) between 35–45 mmHg and facilitate stable end-tidal sevoflurane (ETsevo) concentrations, which was targeted at 2.4%. Once ETsevo stabilized between 2.3 and 2.5% for 15 min, a final ECG recording was obtained. Final recordings during sevoflurane anesthesia were obtained approximately 35 min after opioid administration. All data were obtained prior to the onset of the scheduled elective procedure. Dogs in part two (groups M and H) were administered methadone or hydromorphone as in part one without induction of general anesthesia and ECG recordings were obtained at baseline, 2, 5, 10, and 35 min after opioid administration.

Standard 6-lead ECG recordings were obtained at a paper speed of 25 mm/s. A standard resting ECG filter was used, with a low pass filter of 150 Hz, a line filter of 60 Hz, and an anti-drift system. Once ECG printouts were obtained, HR, PR interval, and QT interval were manually determined in random order by a blinded board-certified veterinary cardiologist (RF) in lead II. Measurements for PR, RR and QT intervals were taken in triplicate, with a measurement from each of three consecutive complexes, and subsequently averaged to obtain the value for the specific time point. The RR interval was used to determine HR. The HR corrected QT interval (QT_c_) was then calculated using the manually obtained QT interval and HR values with Bazett's (QT_cB_), Fridericia's (QT_cF_), and Van de Water's (QT_cV_) formulas (16, 17):

Bazett's formula: QTc_B_ = QT/$\sqrt{60/\text{HR}}$

Fridericia's formula: QT_cF_ = QT/${}^{3}\sqrt{60/\text{HR}}$

Van de Water's formula: QT_cV_ = QT – 87 ^*^ [(60/HR) −1]

### Statistical Analysis

Data were assessed for normality using the Shapiro-Wilk test. The patient variables of body weight and age were compared among all groups using ANOVA. Standard *t*-tests were applied to compare propofol dose, ETCO_2_, ETsevo, and duration of sevoflurane at the time of the last recorded ECG between groups MS and HS. Differences within and between the four treatments (H, HS, M, MS) were determined using a factorial ANOVA [treatment, time (>0), and treatment by time (>0)]. The treatment value at time 0 was a covariate. Pair-wise comparisons were adjusted using the Sidak and Bonferroni methods. All *t*-values and *F*-values were converted to Cohen's *d* effect size to aid interpretation of their clinical relevance. Statistical analysis used SAS version 9.4 (SAS Institute Inc., Cary, NC) and the declaration of statistical significance was *p* < 0.05.

## Results

Data were normally distributed and parametric tests were applied. Patient variables and breed descriptions are listed in Table 1. Body weight (*p* = 0.42) and age (*p* = 0.29) did not differ among treatment groups. Values for ETCO_2_ (*p* = 0.08), ETsevo (*p* = 0.19), and duration of sevoflurane anesthesia at the time of final ECG data collection (*p* = 0.23) did not differ between groups HS and MS.

**Table 1:** Patient variables in dogs randomly assigned to receive either methadone (0.5 mg/kg) or hydromorphone (0.1 mg/kg) IV with electrocardiogram recordings obtained while awake (groups M and H) or while awake and during sevoflurane anesthesia following 15 min with end-tidal sevoflurane stabilized at 2.4% (groups MS and HS, respectively).

	**Methadone**		**Hydromorphone**	
	**MS (*n* = 12)**	**M (*n* = 8)**	**HS (*n* = 12)**	**H (*n* = 8)**
Body weight (kg)	21.4 ± 10.5	18.4 ± 9.2	16.7 ± 11.8 kg	26.0 ± 18.4
Age (year)	3.2 ± 1.5	2.8 ± 2.0	2.0 ± 1.6	2.1 ± 1.5
Breed	Golden Retriever	Mixed Breed Dog	Boston Terrier	Boxer
	Pit Bull Terrier	French Bulldog	American Cocker Spaniel	Australian Shepherd
	Mixed Breed Dog	Mixed Breed Dog	Mixed Breed Dog	Collie
	Yorkshire Terrier	Corgi	Maltese	Great Dane
	Havanese	Mixed Breed Dog	Mixed Breed Dog	Mixed Breed Dog
	Labrador Retriever	Golden Retriever	Cavalier King Charles Spaniel	Pit Bull Terrier
	Mixed Breed Dog	Boxer	Mixed Breed Dog	Great Dane
	Boxer	Pit Bull Terrier	Mixed Breed Dog	Mixed Breed Dog
	Boston Terrier		Havanese	
	Samoyed		German Shepherd	
	Golden Retriever		Mixed Breed Dog	
	Australian Shepherd		Boxer	
Propofol dose (mg/kg)	3.6 ± 1.1	–	3.4 ± 1.1	–
ETCO_2_ (mmHg [kPa])	35.8 ± 3.1 [4.8 ±0.4]	–	38.3 ± 3.4 [5.1 ± 0.5]	–
ETsevo (%)	2.40 ± 0.01	–	2.41 ± 0.01	–
Sevoflurane duration (min)	21.7 ± 3.0	–	20.1 ± 3.3	–

Electrocardiogram variables are displayed in Table 2. Heart rate decreased in all treatment groups at various time points compared to baseline values, with values in HS higher than those in groups M and MS at some time points. The PR interval was prolonged compared with baseline values within each group, with no differences between treatment groups. Values for QT intervals were prolonged compared with baseline values at various time points for each treatment. Corrected QT intervals using different equations were consistently prolonged during sevoflurane anesthesia compared with baseline values. In groups MS and HS, mean values for QT_cV_ during sevoflurane anesthesia were greater than the preceding time point, and greater than values in their respective control groups at the equivalent time point. Fewer differences within and between groups were present with QT_cB_ and QT_cF_, than with QT_cV_. Significant differences in values for all ECG variables occurred between the 10-min time point and the final time point in groups MS and HS only. The only changes in rhythm following opioid administration were bradycardia and respiratory sinus arrhythmias observed with both treatments.

**Table 2:** Electrocardiogram variables obtained in dogs before (Baseline), and 2, 5, and 10 min after the administration of methadone (0.5 mg/kg) or hydromorphone (0.1 mg/kg) IV, as well as either 35 min after administration [groups M (*n* = 8) and H (*n* = 8), respectively] or following 15 min of sevoflurane anesthesia [groups MS (*n* = 12) and HS (*n* = 12), respectively].

		**Time (minute)**				
**Variable**	**Treatment**	**Baseline**	**2**	**5**	**10**	**35/Sevo**
HR (beats/minute)	MS	112 ± 19	90 ± 30^*^	77 ± 19^*^^,^^hs^	76 ± 23^*^^,^^hs^	54 ± 7^*^^,^^§^^,^^hs^
	M	120 ± 21	80 ± 23^*^	83 ± 21^*^^,^^hs^	76 ± 16^*^^,^^hs^	70 ± 12^*^
	HS	115 ± 23	109 ± 27^m^	99 ± 25^*^^,^^m^^,^^ms^	97 ± 23^*^^,^^m^^,^^ms^	71 ± 16^*^^,^^§^^,^^ms^
	H	112 ± 29	104 ± 35	83 ± 20^*^	79 ± 15^*^	74 ± 15^*^
PR interval (ms)	MS	105 ± 12	116 ± 22^*^	121 ± 20^*^	123 ± 18^*^	155 ± 42^*^^,^^§^
	M	92 ± 13	110 ± 13	115 ± 13^*^	119 ± 18^*^	123 ± 18^*^
	HS	100 ± 18	103 ± 17	113 ± 22^*^	114 ± 15^*^	137 ± 25^*^^,^^§^
	H	102 ± 14	110 ± 14	116 ± 12^*^	120 ± 5^*^	115 ± 9^*^
QT interval (msec)	MS	206 ± 14	228 ± 24^*^^,^^hs^	242 ± 23^*^^,^^hs^	247 ± 24^*^^,^^hs^	312 ± 31^*^^,^^§^^,^^m^^,^^hs^^,^^h^
	M	200 ± 16	223 ± 14^*^	232 ± 17^*^	238 ± 13^*^	250 ± 15^*^^,^^ms^
	HS	199 ± 18	206 ± 15^ms^	222 ± 17^*^^,^^ms^	226 ± 24^*^^,^^ms^	279 ± 31^*^^,^^§^^,^^ms^
	H	208 ± 22	219 ± 21	234 ± 11^*^	243 ± 14^*^	248 ± 18^*^^,^^ms^
QT_cB_ (msec)	MS	279 ± 19	273 ± 24	271 ± 22	272 ± 25	295 ± 25^*^^,^^§^
	M	281 ± 16	253 ± 32^*^	270 ± 31	264 ± 21	270 ± 32
	HS	273 ± 23	269 ± 29	281 ± 30	282 ± 27	300 ± 32^*^^,^^§^
	H	280 ± 22	282 ± 30	273 ± 30	276 ± 23	272 ± 29
QT_cF_ (msec)	MS	249 ± 17	255 ± 16	260 ± 17	261 ± 16^*^	302 ± 27^*^^,^^§^^,^^m^
	M	251 ± 13	242 ± 20	256 ± 21	255 ± 14	263 ± 25^ms^
	HS	245 ± 17	246 ± 18	260 ± 19	262 ± 21^*^	293 ± 27^*^^,^^§^
	H	253 ± 18	258 ± 18	259 ± 19	264 ± 15	263 ± 21
QT_cV_ (msec)	MS	245 ± 11	252 ± 17^*^	258 ± 18^*^	259 ± 17^*^	301 ± 26^*^^,^^§^^,^^m^^,^^h^
	M	242 ± 11	239 ± 17	253 ± 16	253 ± 12	261 ± 23^*^^,^^ms^^,^^hs^
	HS	239 ± 14	240 ± 14	253 ± 16^*^	255 ± 20^*^	289 ± 27^*^^,^^§^, ^m^^,^^h^
	H	246 ± 16	251 ± 14	255 ± 15	261 ± 13^*^	260 ± 19^*^^,^^ms^^,^^hs^

* *Significant difference compared to baseline within a group (p < 0.05)*.

§ *Significant difference between last two time points within a group (p < 0.05)*.

m *Significant difference compared to group M at a given time point (p < 0.05)*.

ms *Significant difference compared to group MS at a given time point (p < 0.05)*.

hs *Significant difference compared to group HS at a given time point (p < 0.05)*.

h *Significant difference compared to group HS at a given time point (p < 0.05)*.

## Discussion

The IV administration of both methadone and hydromorphone resulted in notable changes in cardiac conduction. Heart rate was reduced, and PR and QT_c_ intervals were prolonged at various time points following the administration of either opioid in both conscious and anesthetized dogs. Furthermore, general anesthesia increased the magnitude of these changes. The potentiation of these effects during inhalational anesthesia was anticipated based on clinical impressions and the effects of inhalational anesthetics reported in the literature. Currently used inhalant anesthetics, including sevoflurane, are known to prolong QT interval, while propofol does not (18, 19). The effect of anesthesia on these variables may be the result of changes in autonomic tone secondary to the loss of consciousness or more likely direct drug effects on the autonomic nervous system or myocardium given the differing effects of different general anesthetics. Noxious stimulation was not applied in the current study to assess the surgical plane of anesthesia; however, anesthetic depth was deemed to be appropriate in all dogs based on jaw tone and eye position, as well as palpebral reflexes during the data collection period.

In clinical practice, HR is the most readily assessed of the recorded variables, and full mu opioid agonists are known to result in vagally mediated bradycardia (20, 21). While heart rates decreased over time in all groups, HR values were not consistently different between methadone and hydromorphone treatments in conscious dogs, suggesting there was not a clear difference in treatment effect while dogs were awake. However, in dogs undergoing general anesthesia, the addition of sevoflurane further decreased HR with both opioid treatments, with lower HR in methadone treated dogs. This may be a result of differences in dosing equivalency between the two opioids, or due to greater effects of methadone on cardiac conduction and a potential interaction with sevoflurane. Unlike hydromorphone, the chemical structure of methadone resembles that of calcium channel blocking agents (12), resulting in functional L-type calcium channel blockade. This blockade is independent of its mu agonist effects, as morphine does not demonstrate this effect (22). Bradycardia associated with opioid administration is typically readily treatable with anticholinergic administration, and high doses of atropine have been shown to prevent methadone induced bradycardia (21). The additional effect of calcium channel blockade associated with methadone administration may make this intervention less effective compared with other opioids when commonly used clinical doses of anticholinergics are administered, although this has not yet been directly evaluated.

The calcium channel blocking action of methadone would not only affect the sinoatrial (SA) node, slowing depolarization and causing bradycardia, but also act at the AV node slowing conduction and prolonging the PR interval, creating the potential for AV block. While no AV block was noted in the current study, PR interval was prolonged following opioid administration. Interestingly, PR interval was prolonged similarly with both methadone and hydromorphone administration, even with significant differences in HR between groups. This suggests a discrepancy in the influence of methadone and hydromorphone on SA and AV nodal activity, which may be due to differences in expression of the funny pacemaker current in the different nodal cells (23). These results suggest that either hydromorphone has proportionally greater suppression at the AV node compared to the SA node, or that methadone has less AV nodal influence and a stronger effect on pacemaker cells within the SA node.

To appropriately interpret changes in QT interval, the value must be corrected for HR. Several equations have been proposed, with Van de Water's formula being most strongly recommended for use in dogs (17). The different formulas applied to calculate QT_c_ in the current study all yielded different results, with Van de Water's formula resulting in significant QT_c_ prolongation with both methadone and hydromorphone administration. Prolongation in QT interval is a documented complication of methadone administration in the human literature, increasing the risk for developing both early after depolarizations and the malignant arrhythmia, torsades de pointes (7). The magnitude of QT interval prolongation with methadone in human patients is dose-dependent; however, even low dose methadone administration increases repolarization time and does not eliminate the risk of subsequent arrhythmias (7).

The mechanism of QT interval prolongation with methadone and other pharmacologic agents has been elucidated in human *in vitro* assays, and has been the focus of several reviews (24–26). Methadone blocks cardiac potassium channels and subsequently interrupts the delayed rectifier potassium current. The reduction in potassium efflux prolongs the action potential and delays ventricular repolarization, which manifests as a prolonged QT interval on the electrocardiogram (26). Additionally, the injectable formulation of methadone contains the preservative chlorobutanol which acts synergistically with methadone to block the efflux of potassium ions, further enhancing this adverse effect (7). In humans, the affected potassium channel is encoded by the human ether-a-go-go related gene (hERG) (25). While the corresponding gene has not been characterized in small animals, other known hERG blockers also cause QT prolongation when administered to conscious dogs (27), suggesting that the mechanism is similar.

Hydromorphone has not yet been identified as an agent that prolongs QT interval, and is a novel finding in the current study. The mechanism for QT interval prolongation with hydromorphone is currently unknown. Whether this effect is unique to dogs, or is also a consideration in human medicine warrants further investigation, as does the effect of dose and route of administration. While the magnitude of QT interval prolongation was greater following methadone than with hydromorphone administration during sevoflurane anesthesia, the mean prolongation for both treatments considerably exceeded the 2005 U.S Department of Health and Human Services industry guidelines for QTc prolongation (28), which state that QTc prolongation >20 ms substantially increases arrhythmogenic risk in humans. While the degree of prolongation that increases arrhythmogenic risk has not been clearly defined in dogs, the effect of pharmaceutical agents on QT interval is of particular concern in patients with inherited QT prolongation, a condition that has been reported in a group of English Springer Spaniels (29). Pharmaceutical QT interval prolongation may also be relevant in the presence of electrolyte and acid-base abnormalities, including hypercalcemia, hypermagnesemia, and hypokalemia, which are associated with prolongation of the QT interval (30).

There are some considerations when interpreting the data from the current study. A clinical population of dogs was evaluated, which introduced more variability than a homogeneous population, but provided more representational data of a broader clinical population. Of note, brachycephalic dogs are generally considered to have higher resting vagal tone and were included in the study. Only brachycephalic dogs with regular sinus rhythm were enrolled, and those with respiratory sinus arrhythmias or other arrhythmias were excluded. Additionally, using corrected QT intervals removed any influence of vagally induced heart rate changes on QT interval for interpretation. Interestingly, the enrolled brachycephalic patients had values both higher and lower than mean values for the variables evaluated. Despite differences in the breed, age, and sex of the dogs evaluated, which would be expected to increase variability of the results, the findings were still statistically significant, indicating a clear clinical effect of the opioids administered.

An additional consideration is dose selection. There are no studies that directly compare the sedative effects or analgesic efficacy of hydromorphone and methadone in dogs, making it challenging to select equivalent doses. In dogs, the clinically administered dose range for hydromorphone is 0.05–0.15 mg/kg, while the range for methadone is 0.1–1.0 mg/kg (31). The doses in the current study were selected to simulate pre-anesthetic doses, and to evaluate doses that may be used clinically and have the potential to increase arrhythmogenicity. Differing levels of sedation can influence autonomic tone and ECG variables. While formal scoring was not performed, the sedative effects of each opioid appeared very similar, with all dogs displaying mild sedation and voluntarily remaining in lateral recumbency throughout the experimental period with only minor responses to interactions with the investigators. Route of administration may also differ from routine pre-medication, which is often administered IM, but due to variability in IM absorption, IV administration was selected to standardize the timeframe of ECG effects. The current study demonstrated that both methadone and hydromorphone administration created proarrhythmogenic environment; however, additional studies evaluating different opioid doses and routes of administration are required to characterize these changes.

The study also has specific limitations to note. Blood pressure is intrinsically linked to heart rate and autonomic tone, but was not reported in the current study as the aim was to evaluate drug-induced ECG alterations. Neither of the opioids evaluated have clinically relevant vasoactive effects, however changes in heart rate would have altered cardiac output and subsequently blood pressure, and this information would be informative about the overall clinical effects of these opioids. Additionally, cardiac function of dogs in the study was not comprehensively defined as they did not receive echocardiographic evaluations prior to enrollment, and occult heart disease cannot be excluded. Thus, the study findings apply to young, clinically healthy dogs, as those with clinical cardiac disease and greater arrhythmogenic potential were not evaluated. Despite these limitations, the study identified changes in cardiac electrical conductivity resulting from methadone and hydromorphone administration, highlighting the importance of considering the arrhythmogenic effects of these agents.

## Conclusions

Both methadone and hydromorphone altered HR, PR interval, QT interval, and corrected QT interval when administered IV, with greater effects observed during sevoflurane anesthesia. Prolongation of the QT interval with both hydromorphone and methadone exceeded industry safety guidelines for human medications. While no malignant arrhythmias were observed in the current study evaluating healthy dogs, the arrhythmogenic potential of these agents in dogs with cardiac disease, particularly methadone, should be considered, and warrants further investigation.

## Data Availability Statement

All datasets generated for this study are included in the article/supplementary material.

## Ethics Statement

This animal study was reviewed and approved by IACUC of the University of Illinois at Urbana-Champaign. Written informed consent was obtained from the owners for the participation of their animals in this study.

## Author Contributions

SK: study design, data acquisition and interpretation, and preparation of manuscript. RF: study design, data acquisition and interpretation, and manuscript revision. KK and LG: study design, data acquisition, and manuscript revision. SC-P: study design and manuscript revision. DS: statistical analysis and manuscript revision. All authors contributed to the article and approved the submitted version.

## Conflict of Interest

The authors declare that the research was conducted in the absence of any commercial or financial relationships that could be construed as a potential conflict of interest.
